# Compressive Performance of CFRP Ring-confined Circular LSCFST columns

**DOI:** 10.1371/journal.pone.0357309

**Published:** 2026-09-08

**Authors:** Zhiping Deng, Qiuyu Liu, Yujiao Qin, Caisen Wang, Jiongfeng Liang, Haizhong Zhang

**Affiliations:** 1 Jiangxi Provincial Underground Engineering Risk Digital Monitoring and Control Research Center, East China University of Technology, Nanchang, China; 2 School of Civil & Architecture Engineering, East China University of Technology, Nanchang, China; 3 Nanchang Architecture Science Institute Co., Ltd., Nanchang, China; 4 College of Architecture and Civil Engineering, Beijing University of Technology, Beijing, China; 5 Eco-Science Course, Faculty of Agriculture, Yamagata University, Yamagata, Japan; King Mongkut's University of Technology North Bangkok, THAILAND

## Abstract

To explore the disposal method of lithium slag (LS), 20 columns were tested with three parameters, LS replacement rate, carbon fiber reinforced polymer (CFRP) ring spacing, and CFRP layer number, respectively. The conclusion is that the lithium-slag concrete-filled steel tube (LSCFST) stub columns subjected to CFRP circumferential constraints undergo a progressive failure process, ultimately resulting in the steel tube bucking; By the increasing LS replacement rate, the bearing capacity of columns shows an uptrend, while their ductility deteriorates. And the columns with 20% of replacement rate show better load capacity than other specimens. Compared to ring spacing, the adding layers of CFRP has a greater enhancement on the bearing capacity and ductility of columns. Finally, an ultimate strength formula for columns is established and its accuracy is verified.

## 1. Introduction

At present, concrete-filled steel tube (CFST) columns are extensively employed in high-rise structures. As a type of composite column, the steel tube works in cooperated with the concrete poured into it to get better loading capacity, compared with the single steel tube column [1–4]. The steel tube provides the constraining stress on the concrete, thereby delaying its cracking. And the core concrete in turn prevents the premature local buckling of the steel tube. According to the cross-section form, square and round are the most common cross-sectional forms of CFST columns. Circular CFST columns are extensively utilized on bridges, high-rise buildings, and ultra-high buildings due to their good mechanical properties and simple construction process [5–7].

To solve the problem of waste environmental pollution, it is necessary to fully utilize industrial by-products as a new green building materials [8–10]. Many scholars have found that the lithium slag (LS) waste, produced by calcining Spodumene, is a high-quality concrete admixture. And mechanical properties of ordinary concrete, to some certain extent, can be improved by LS powder [11]. Amin et al. [12] have investigated transport properties of concrete by replacing supplementary cementitious material with LS at 20%, 40%, and 60% levels. And results showed that the compressive strength of concrete with 20% to 40% of LS content was enhanced, and its water infiltration and porosity were also reduced. In addition, the LS concrete with 40% content had a 18.34% higher compressive strength than the control group at 180 days. Zhang et al. [13] discussed the changing content of LS and limestone powder (LP) on the performance of ultra-high performance concrete (UHPC). It was found that SO_4_^2-^ was supplemented by LS for UHPC. In addition, when the total replacement rate is 30%, the optimal ratio of LS to LP in UHPC is 2:1, which can achieve the best performance.

**Fig 1:**
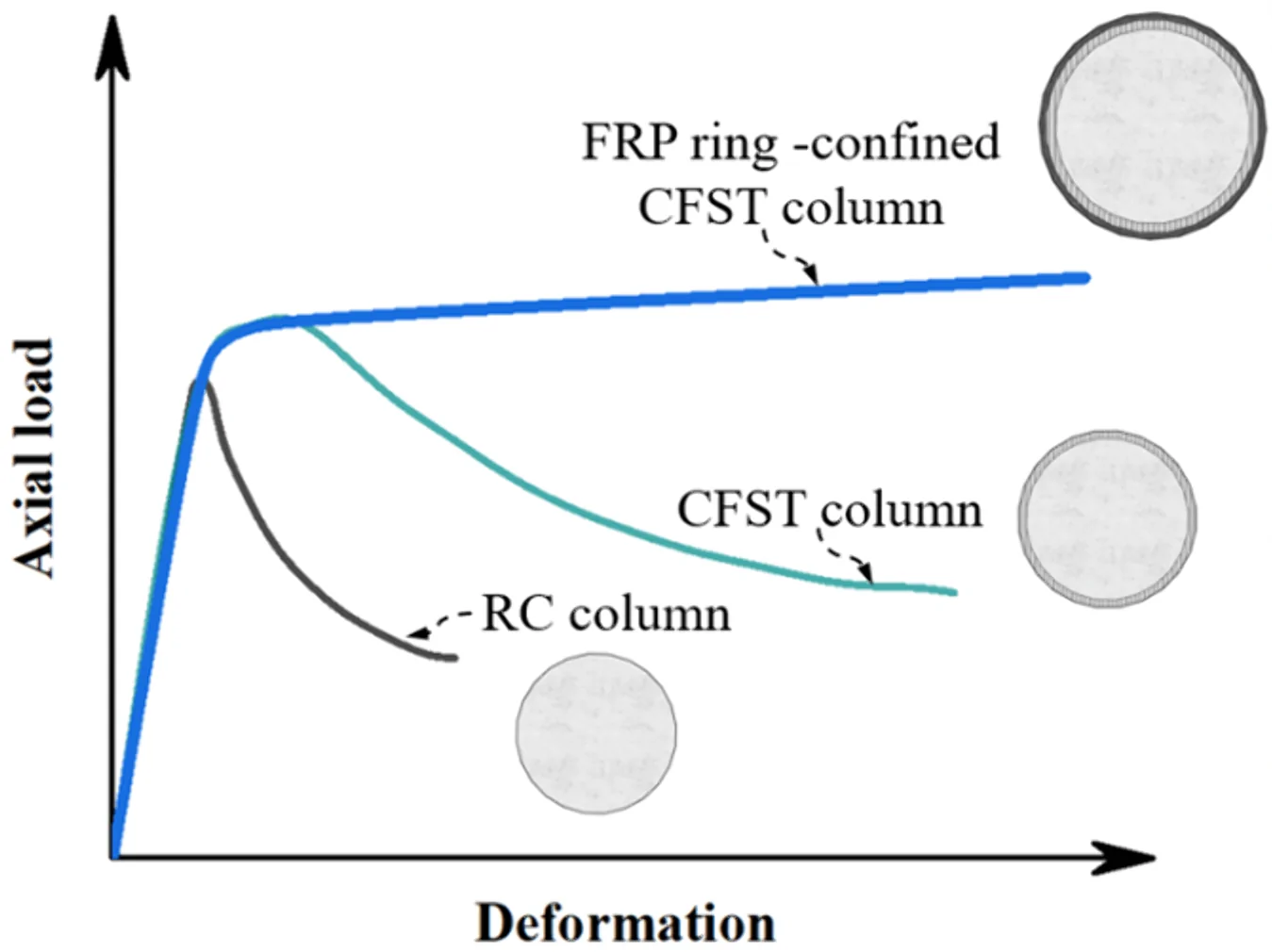
Columns with different confinement levels.

Fiber materials are lightweight yet highly durable, making them widely used in various fields [14–17]. Moreover, composite materials in the form of FRP sheets and tubes have been proven to be an effective strengthening method for concrete, and researchers have proposed the concept of using FRP confinement to reinforce CFST columns [18]. As shown in Fig 1, Wang et al. [19] analyzed the behavior of CFRP-CFST members under combined compression-bending-torsion loading and noted that the slope of the second segment of the load-deformation curve increased significantly. It is found that the column ductility was effectively improved by wrapped CFRP, and its energy absorption capacity also rose with the number of CFRP layers. Moreover, there are two types of reinforcement type: partial wrapping strengthening technology, and full wrapping strengthening technology [20–24]. Miao et al. [25] conducted the compression test on CFST columns confined by CFRP or welding steel strips, and the findings showed that the ultimate load of the CFST columns improved by 34.31% due to the reinforcement provided by the FRP.

The use of lithium slag as a partial cement replacement can reduce cement consumption and promote the recycling of industrial solid waste. When combined with CFRP ring confinement, LSCFST columns may provide an efficient structural solution with both environmental and mechanical benefits

It mainly reports in this paper that the investigation of the axial behavior of CFRP ring-confined circular LSCFST columns with parameters including LS content, CFRP ring spacing, and CFRP wrapping layers. And the failure mode, bearing capacity and ductility are analyzed. Additionally, a method for predicting their ultimate strength is proposed.

## 2. Experimental procedure

### 2.1. Specimen design

The experimental programme was designed to investigate three main parameters: LS replacement ratio, CFRP ring spacing and number of CFRP layers. These parameters were selected to evaluate the influence of the concrete core material, the distribution of external confinement and the confinement intensity on the axial behaviour of circular LSCFST columns.

**Fig 2:**
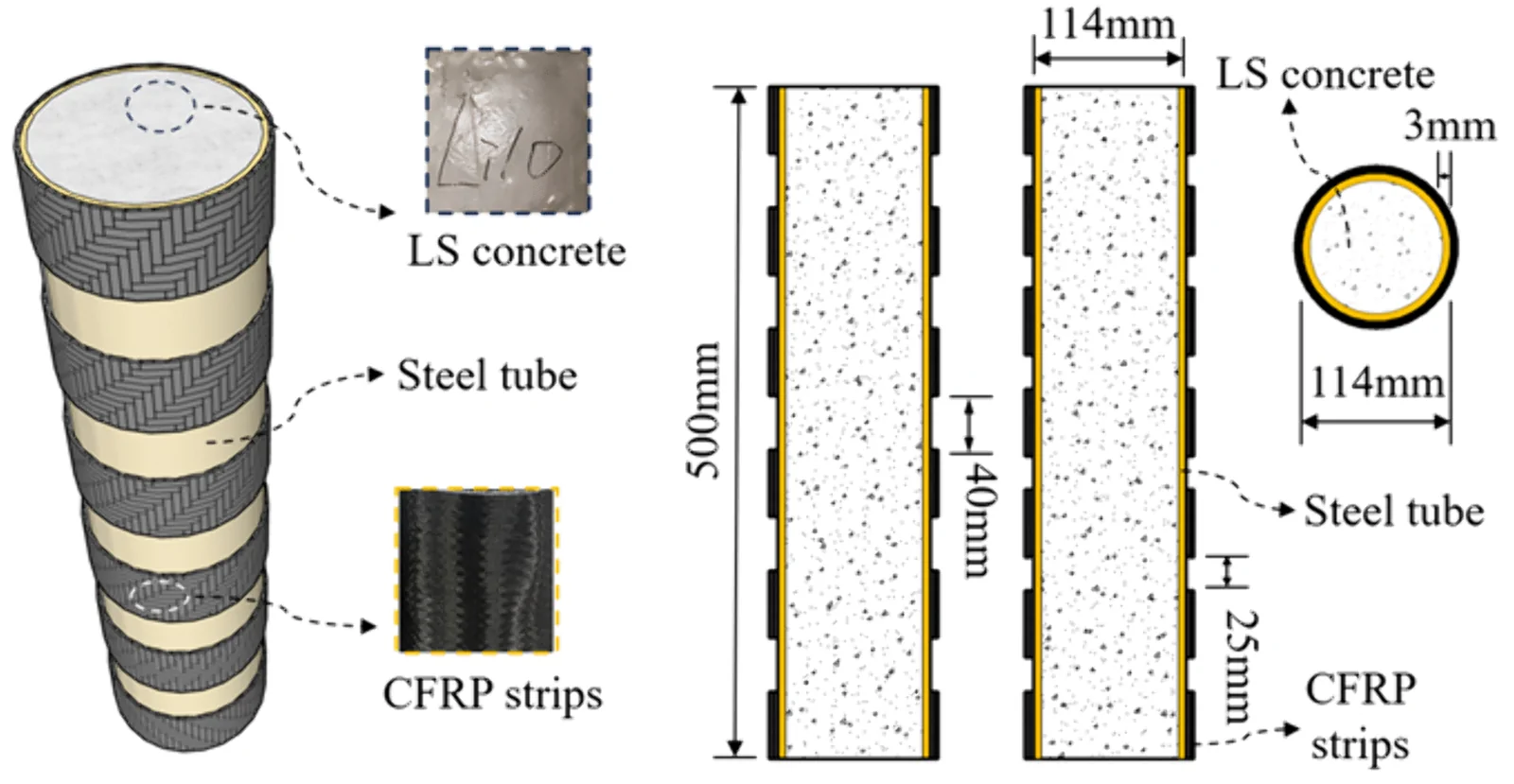
The schematic view of test columns. (a)Steel tube polishing. (b)Concrete pouring. (c)CFRP strips wrapping. (d)Specimens curing.

Specimens are 20 columns, including (i) 1 pair of CFST columns; (ii) 4 pairs of CFRP confined CFST columns; (iii) 5 pairs of CFRP confined LSCFST columns. Each pair means two columns with identical parameters. The schematic diagram of the LSCFST column wrapped with CFRP rings is shown in Fig 2. All columns are fabricated from the same batch of steel tubes with the thickness (*t*_s_) of 3 mm, height of 500 mm, outer diameter (*d*_s_) of 114 mm. The ratio of height to minimum lateral dimension is less than 5. CFRP ring widths (*b*_f_)are 50 mm ^[22,23]^. Different replacement rates (i.e., 0%, 10%, and 20%), Two types of CFRP ring spacing (*s*_f_) (i.e., 25 mm, and 40 mm), and Four CFRP layers numbers (i.e., 0, 1, 2, and 3) are adopted in the experiment.

The detailed information of the columns is listed in Table 1. Each column is labeled based on the following rules: (i) letters of PW represents the partial wrapping scheme, while the number denotes the spacing of the CFRP ring; (ii) The letter F represents the CFRP, and the number denotes the count of layers; (iii) The number in parentheses represents the number of duplicate columns; (iv) Individual numbers represent the replacement rate of LS. For example, PW25-F2 (1) −10 is the first repeated column, with a CFRP ring spacing of 25 mm. And its number of CFRP layers is 2, its replacement rate of LS is 10%. Both of two CFST columns are given a two-part name, with the first part being CFST and the last part denoting the numerical order of duplicated samples.

**Table 1:** Details of columns.

Specimen	Steel tube		Concrete		CFRP		
	$d_{s}$ (mm)	$t_{s}$ (mm)	LS replacement rate (%)	${f_{co}}^{\prime }$ (MPa)	$s_{f}$ (mm)	$t_{f}$ (mm)	$\rho_{f}$ (%)
CFST1	114	3	0	32.1	——	——	0
CFST2	114	3	0	32.1	——	——	0
PW40-F1(1)-0	114	3	0	32.1	40	0.167	0.773
PW40-F1(2)-0	114	3	0	32.1	40	0.167	0.773
PW40-F3(1)-0	114	3	0	32.1	40	0.501	2.319
PW40-F3(2)-0	114	3	0	32.1	40	0.501	2.319
PW25-F1(1)-0	114	3	0	32.1	25	0.167	1.237
PW25-F1(2)-0	114	3	0	32.1	25	0.167	1.237
PW25-F3(1)-0	114	3	0	32.1	25	0.501	3.711
PW25-F3(2)-0	114	3	0	32.1	25	0.501	3.711
PW25-F2(1)-10	114	3	10	34.0	25	0.334	2.474
PW25-F2(2)-10	114	3	10	34.0	25	0.334	2.474
PW40-F2(1)-10	114	3	10	34.0	40	0.334	1.546
PW40-F2(2)-10	114	3	10	34.0	40	0.334	1.546
PW40-F1(1)-10	114	3	10	34.0	40	0.167	0.773
PW40-F1(2)-10	114	3	10	34.0	40	0.167	0.773
PW40-F1(1)-20	114	3	20	35.6	40	0.167	0.773
PW40-F1(2)-20	114	3	20	35.6	40	0.167	0.773
PW40-F1(1)-30	114	3	30	33.2	40	0.167	0.773
PW40-F1(2)-30	114	3	30	33.2	40	0.167	0.773

Note: $d_{s}$ —Outer diameter of steel tube; $t_{s}$ —Steel tube thickness; ${f_{co}}^{\prime }$ —Unconfined concrete strength; $s_{f}$ —CFRP strip spacing; $t_{f}$ —CFRP thickness; $\rho_{f}$ —CFRP volume ratio ($\frac{4t_{f}b_{f}}{d_{c}s_{f}}$); $b_{f}$ —CFRP ring width; $s_{f}$ —Centre-to-centre distance between two adjacent CFRP rings; N.A. —Not applicable.

**Fig 3:**
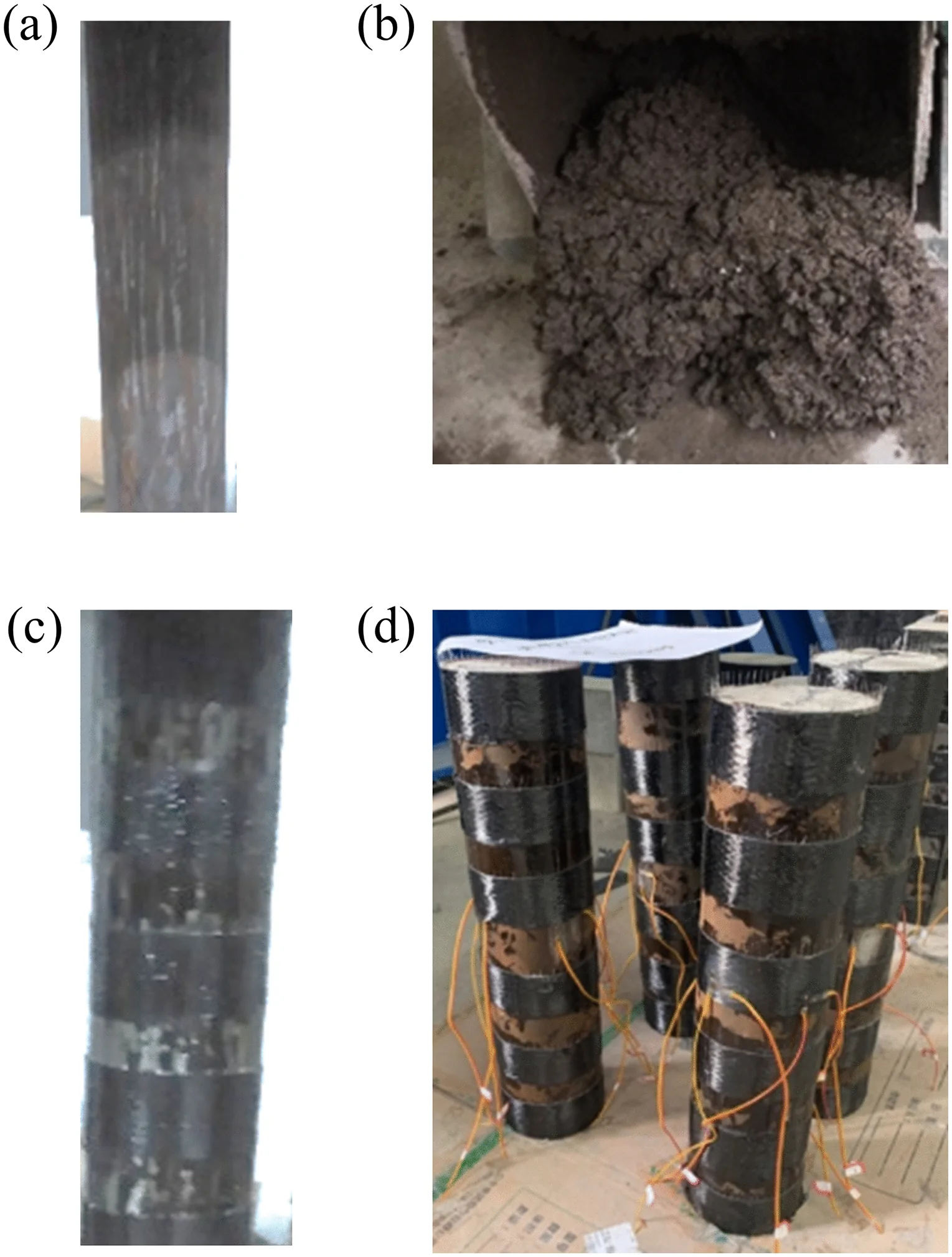
Process of fabricating CFRP confined LSCFST columns.

Samples of LSCFST columns were produced by pouring LS concrete into circular steel tubes. The concrete used was the same batch of LS concrete prepared by replacing cement with LS in a predetermined proportion. Then samples were cured in air for 28 days, concrete blocks with a size of 150 mm × 150 mm × 150 mm were cured in the same time for testing the compressive strength exhibited by LS concrete. CFRP strips were applied onto the outer surface of steel tubes layer by layer after 28 days of curing. And the first strip was located at the middle of the column, then two ends of the column were wrapped in sequence, as shown in Fig 3. The length of the overlapping part was 100 mm, ensuring that the circumferential constraint of CFRP rings was fully utilized [24, 25].

### 2.2. Material

Steel with a strength grade of Q235 was adopted for steel tubes. And the tensile testing of steel samples was conducted according to the specification AS 1391–2007 [26]. The ultimate strength ($f_{y}$), yield strength ($f_{u}$), and elastic modulus ($E_{s}$) are 359MPa, 258MPa, and 2.01 × 105MPa, respectively.

The properties of the CFRP strip are listed as follows: tensile modulus of elasticity ($E_{t}$), the tensile strength ($\sigma_{b}$), and elongation are 2.4 × 105MPa, 3542MPa and 1.6%, respectively.

**Fig 4:**
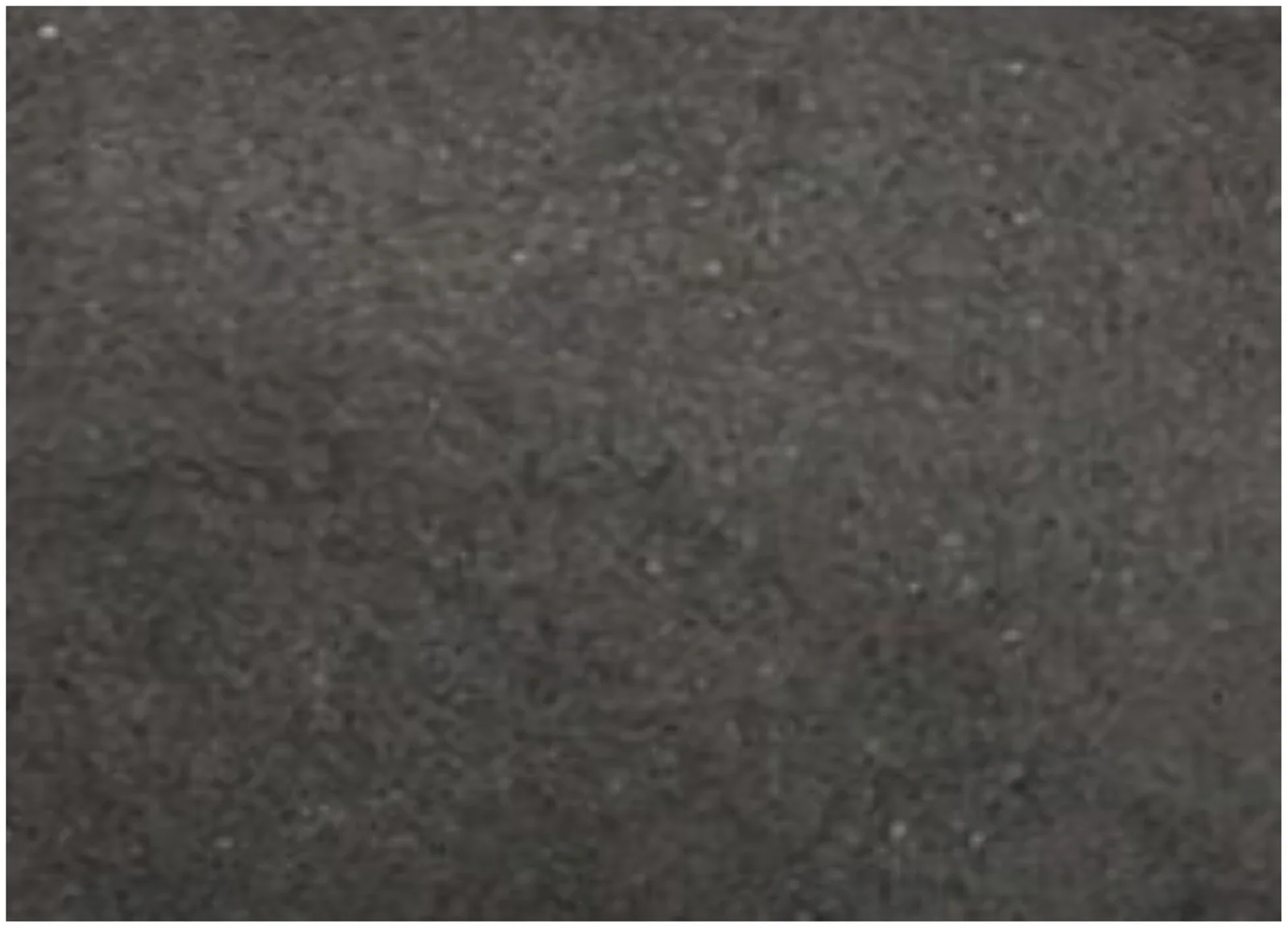
The LS powder appearance.

**Fig 5:**
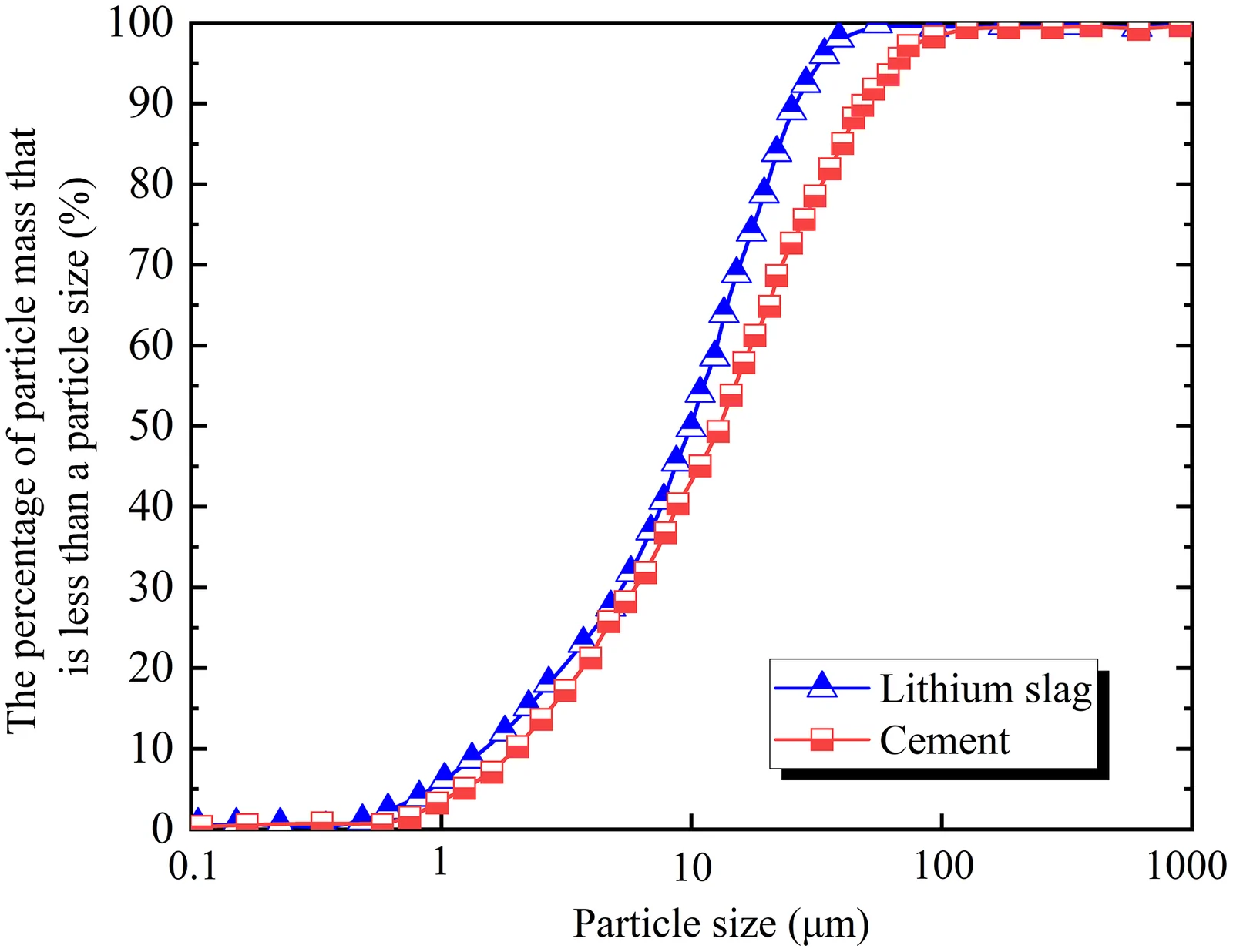
Particle size cumulative distribution curves.

**Fig 6:**
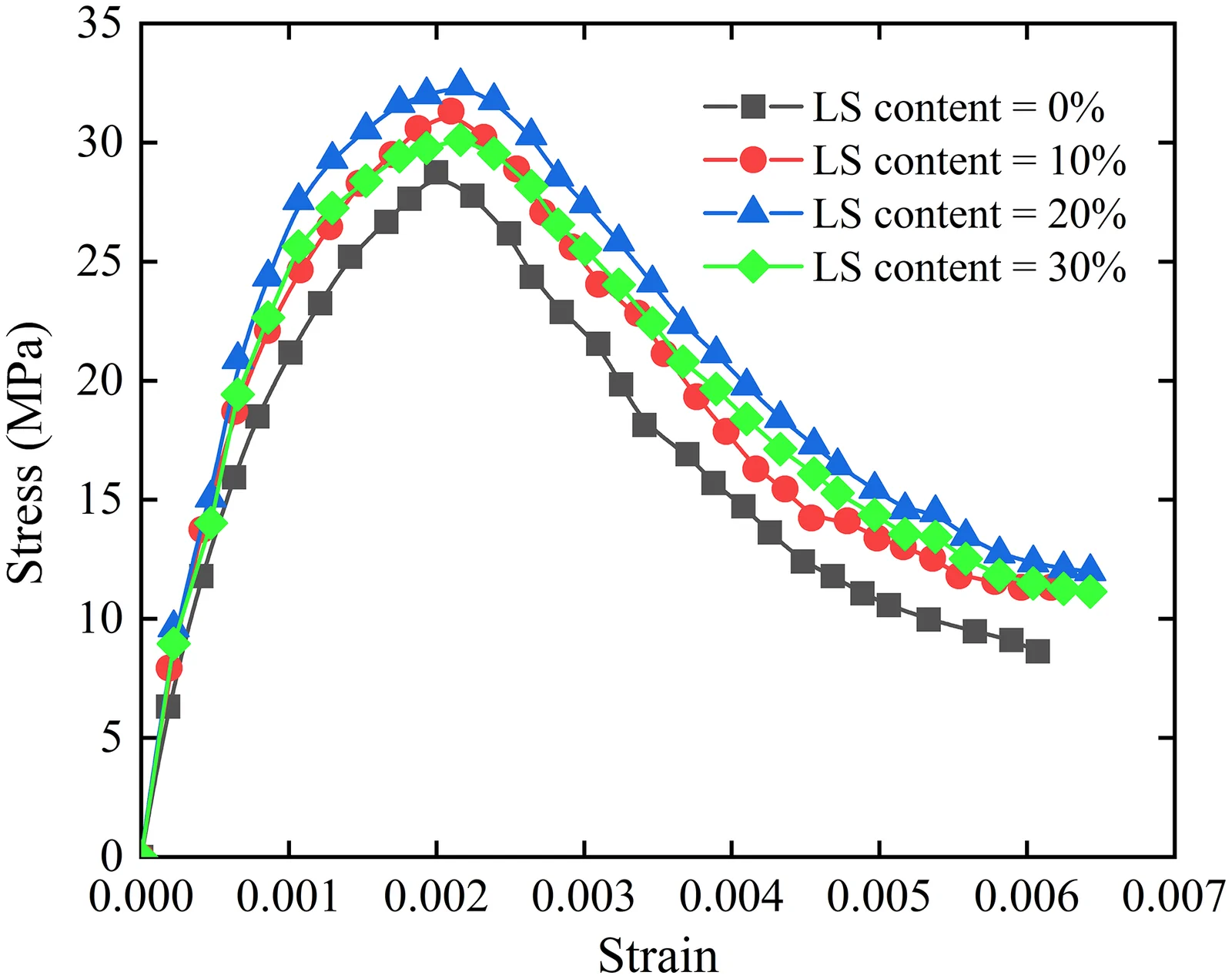
Stress-strain curves of concrete with different LS content.

LS concrete was mixed strictly according to the code JGJ55–2011 [27] to maintain its strength with grade C30. Only the cement was replaced by LS powder in proportion to mass. Table 2 shows mixture proportions of LS concrete applied on specimens. And Fig 4 shows the appearance of LS powder. Fig 5 shows the cumulative particle size distribution curves for cement and LS. Fig 6 shows the stress-strain behavior for LS concrete. It shows that the slope of the ascending curve steepens as the replacement ratio increases. And it indicates that the lithium slag within 20% can react with cement to form C-S-H, which can be filled inside the concrete to compensate for natural defects such as microcracks, thereby improving the ductility of concrete and delaying its failure.

**Table 2:** Mixture proportions of LS concrete.

Strength grade	LS content (%)	Water-cement ratio	Mixture proportion (kg/m^3^)				
			Water	Cement	LS	Sand	Stone
C30	0	0.40	180	450	0	539	1257
C30	10	0.44	180	405	45	539	1257
C30	20	0.46	180	390	90	539	1257
C30	30	0.57	180	315	135	539	1257

Tables 3 and 4 present the chemical composition and physical properties of the LS. The measured chemical composition of lithium slag shows relatively high contents of SiO2, Al2O3 and SO3, and its particle-size distribution is close to that of cement. Therefore, a moderate amount of lithium slag may improve the compactness of the cementitious matrix through micro-filling and secondary hydration/pozzolanic reactions, thereby increasing the compressive strength of the core concrete.

**Table 3:** Chemical composition list of cement and lithium slag (wt%).

Material	SiO_2_	CaO	Al_2_O_3_	SO_3_	TiO_2_	Fe_2_O_3_	MgO	Other
Lithium slag	45.90	9.70	19.3	5.97	2.20	1.23	1.10	14.60
Cement	61.62	21.01	4.55	2.38	0.07	3.43	1.27	5.67

**Table 4:** Physical properties of lithium slag.

Performance	Bulk density (kg/m^3^)	Apparent density (kg/m^3^)	Specific surface area (kg/m^3^)	Moisture content (%)	Water absorption (%)
Lithium slag	1065	1913	763	2.16	3.67

### 2.3. Test setup

**Fig 7:**
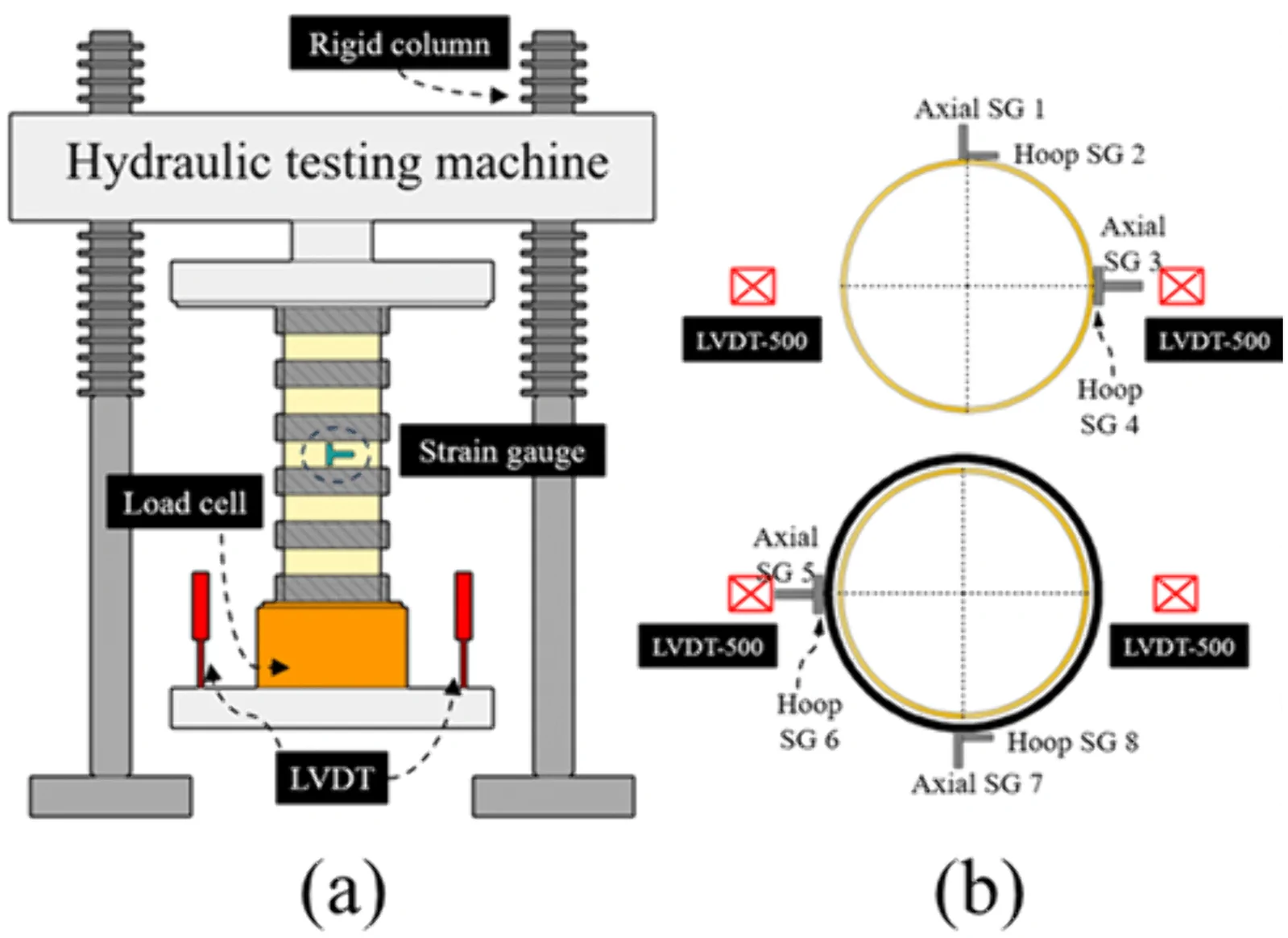
(a) Schematic diagram of the equipment; (b) Arrangement of strain gauges. (a) CFST (b) Partial fracture of CFRP ring (c) Complete fracture of CFRP ring.

A 300t hydraulic testing machine was employed for the tests, as depicted in Fig 7(a). For the CFST columns, totally 4 strain gages (SG) were placed on the surface of the steel tube in the longitudinal and circumferential directions. And there were 8 SGs arranged on the CFRP ring-confined column. In addition, two LVDTs are utilized to measure the axial deformation of tested columns, as shown in Fig 7 (b). The displacement-control method was applied, and its loading rate was set to 0.5 mm/min. All data was recorded by a computer-connected collector. And the test was kept ongoing until the load value is fell to 85% of the peak load.

## 3. Experimental results and discussions

### 3.1. Failure modes

**Fig 8:**
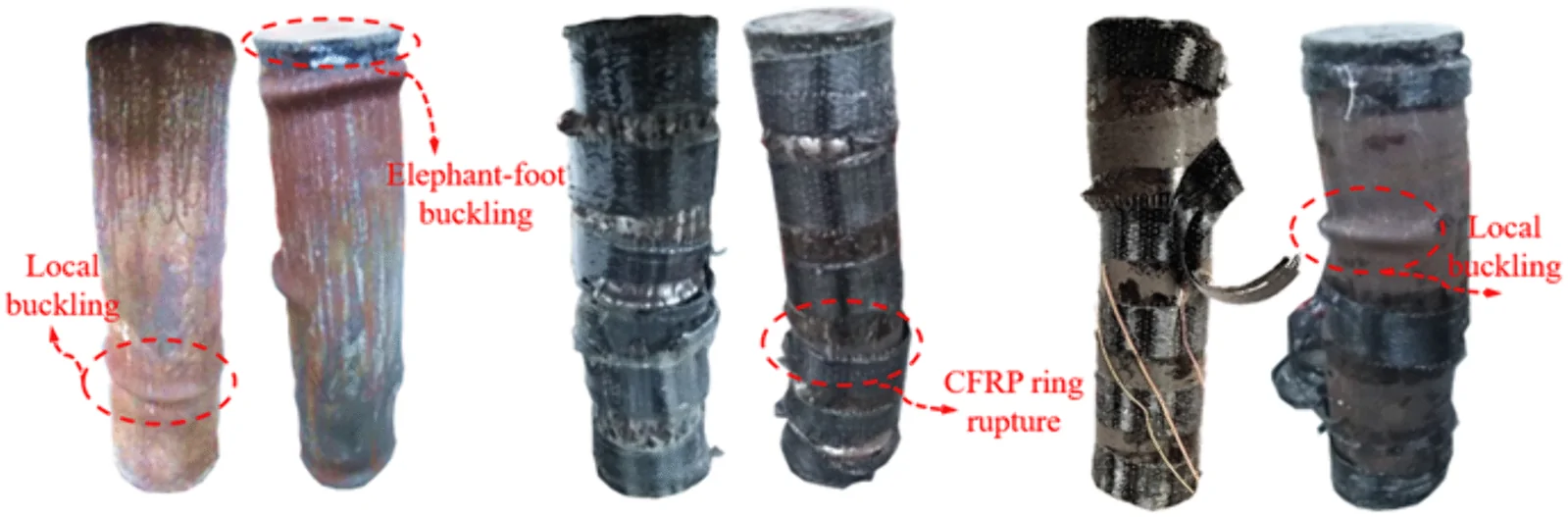
Failure mode.

**Fig 9:**
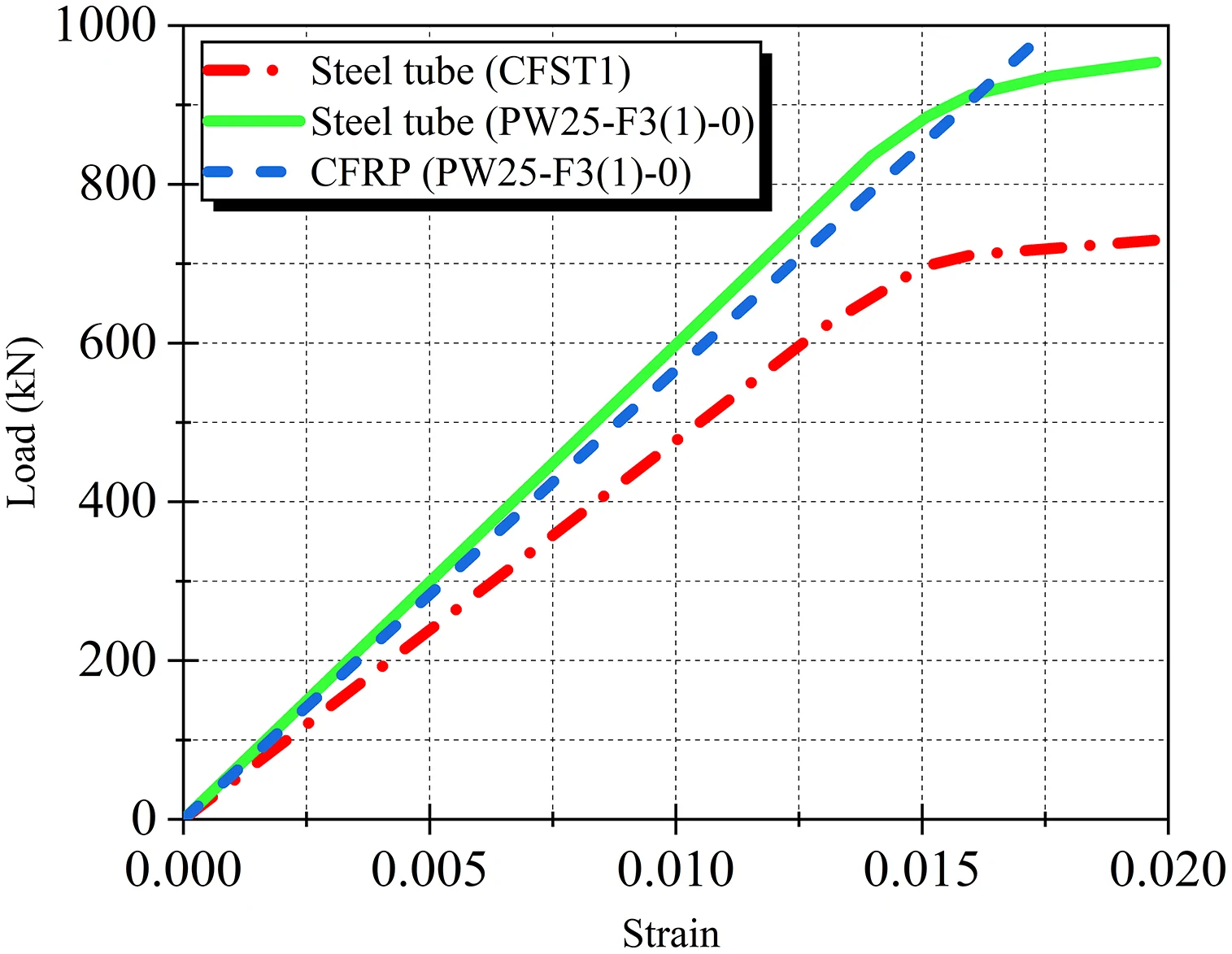
Surface strains of steel tube and CFRP.

For CFST columns, the elephant-foot buckling appeared at both ends of columns and outward bucklings were also observed at the middle of columns, as shown in Fig 8 (a), which is consistent with other literatures [28]. For CFRP ring-confined columns, total of them were failed due to serious local buckings of steel tubes followed by CFRP strips tearing. And the fracture of CFRP strips usually occurred at the middle height of columns, emitting an explosive sound. Failure modes of partial fracture and complete fracture of CFRP strips are shown in Fig 8 (b) and (c). Overall, a progressive failure exhibited on CFRP ring-confined LSCFST columns. And the first rupture of CFRP rings was referred as initial failure state of the column, the last CFRP rings rupture was referred as the total failure state. Based on the loading test, following results can also be obtained: (i) circumferential tensile strains on the surface of LSCFST columns is lower than those of CFST columns due to the constraint of CFRP rings, so LSCFST columns confined with CFRP exhibit better loading capacity than CFST columns, as shown in Fig 9, (ii) more significant deformation and bucklings usually appeared on LSCFST columns with larger spacing or less layers of CFRP.

The CFRP rings provided effective circumferential confinement to the steel tube and concrete core. This confinement reduced lateral expansion, delayed local buckling of the steel tube and postponed the development of severe damage. Consequently, the CFRP ring-confined LSCFST columns exhibited higher axial load-carrying capacity and improved deformation capacity compared with unconfined CFST columns.

### 3.2. Axial load-strain curves

**Fig 10:**
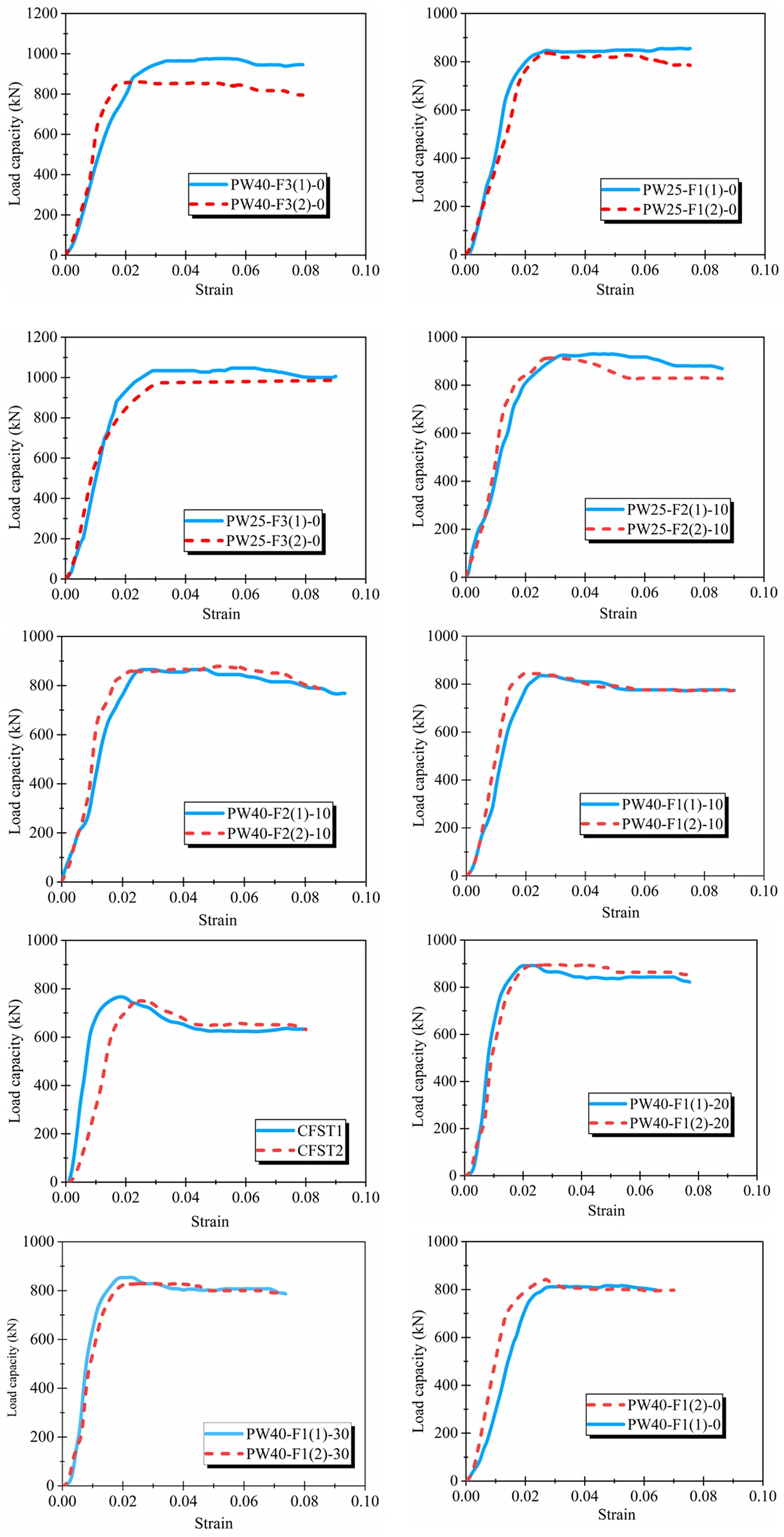
Load-strain curves.

**Fig 11:**
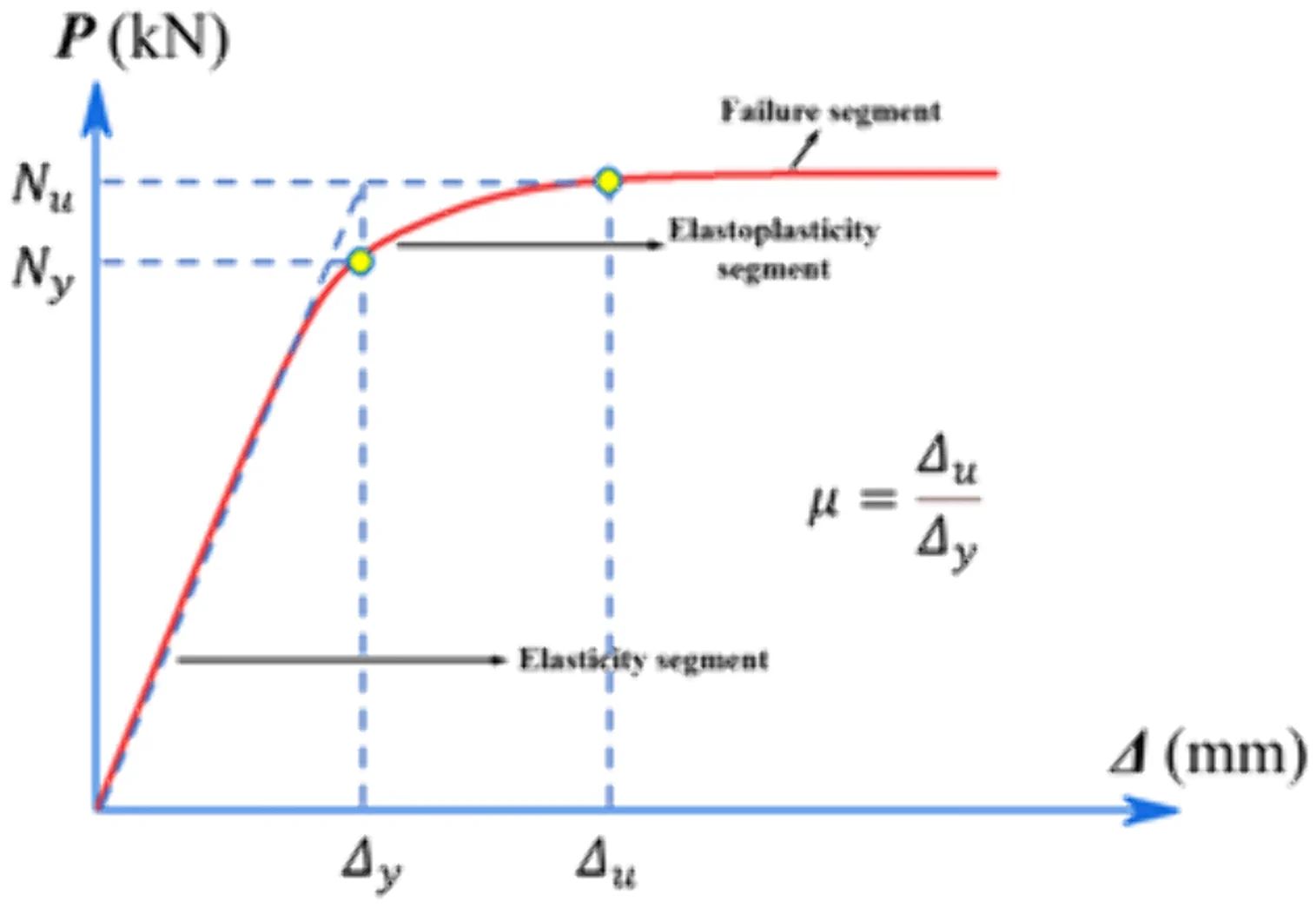
Typical load-strain curves. (a) PW40-F1(1)-0, (b) PW25-F1(1)-0, (c)PW40-F1(1)-10, (d) PW40-F2(1)-10.

The average readings of two longitudinal strain gauges on the surface of steel tubes are regarded as the strain value. The strain value is axial deformation divided by column length (500 mm). Fig 10 shows the load-strain curves of 20 columns. It can be seen that curves of columns can be categorized into three distinct phases: elastic stage, elastic-plastic stage, and failure stage, as shown in Fig 11 [29]. When the loading test begins, columns undergo elastic deformation, characterized by a linear relationship between load and displacement. As loading increases, plastic deformation begins to occur, and the axial deformation of columns increases rapidly. Continuing to load, CFRP strips are widely torn open in the failure stage. At this time, multiple buckling occurs on the steel tube, curves of columns eventually become flat.

**Fig 12:**
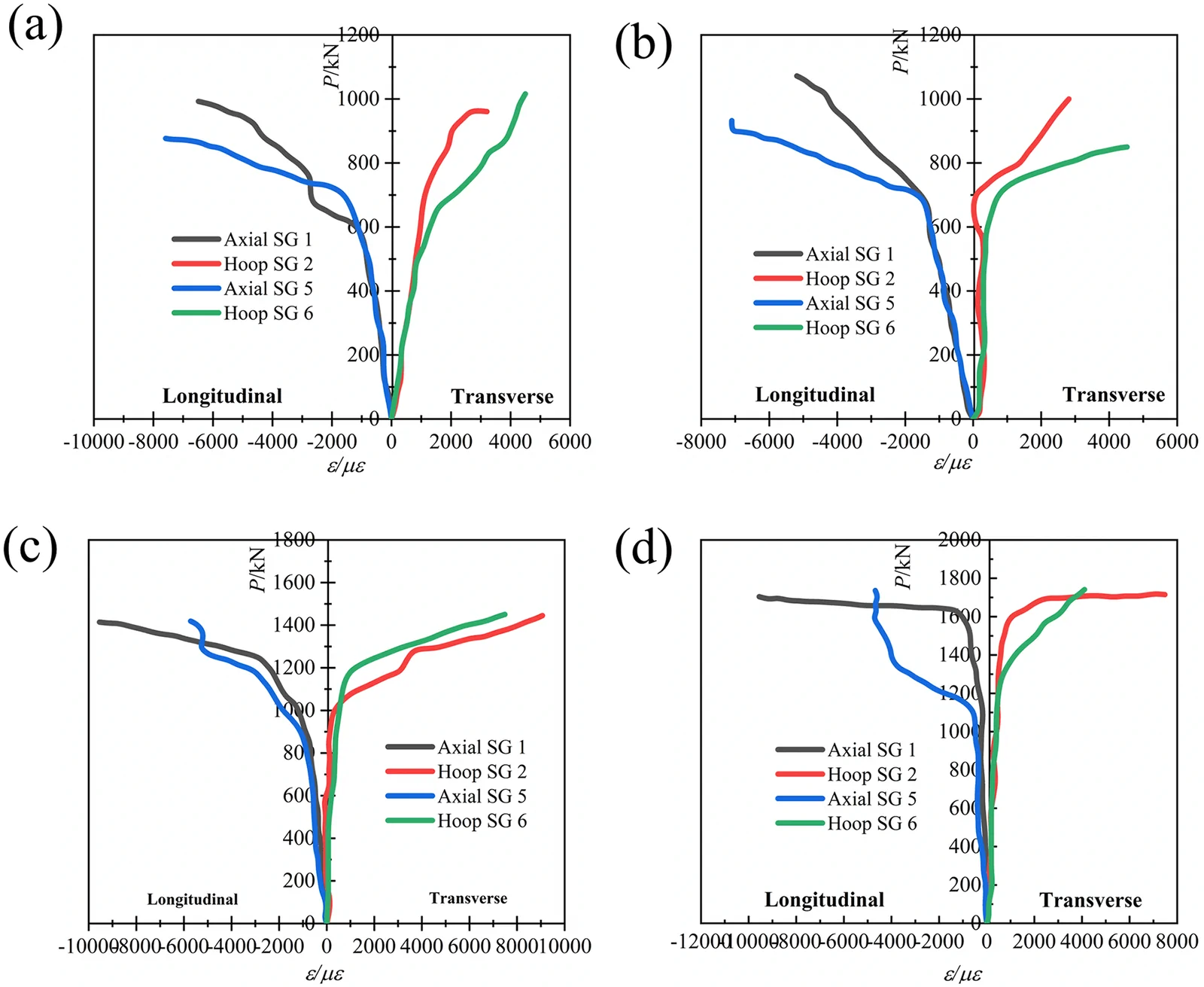
Typical strain distribution of columns.

Fig 12 illustrates the representative strain behavior of the tested columns. Within the elastic range, strain varied linearly with load and increased slightly. Upon entering the elastic-plastic working stage, the slope of the curve diminished and the growth rate of strain was rapid. In the process of axial compression test, it becomes evident that the longitudinal strain of the column was usually greater than the transverse strain, and in the elastic-plastic stage, the growth of the strain of CFRP sheet was significantly greater than that of the steel tube. These findings indicate that the enhancement of CFRP sheets provided lateral confinement for LSCFST columns and delayed the local buckling of the steel tube.

### 3.3. Dilation properties

**Fig 13:**
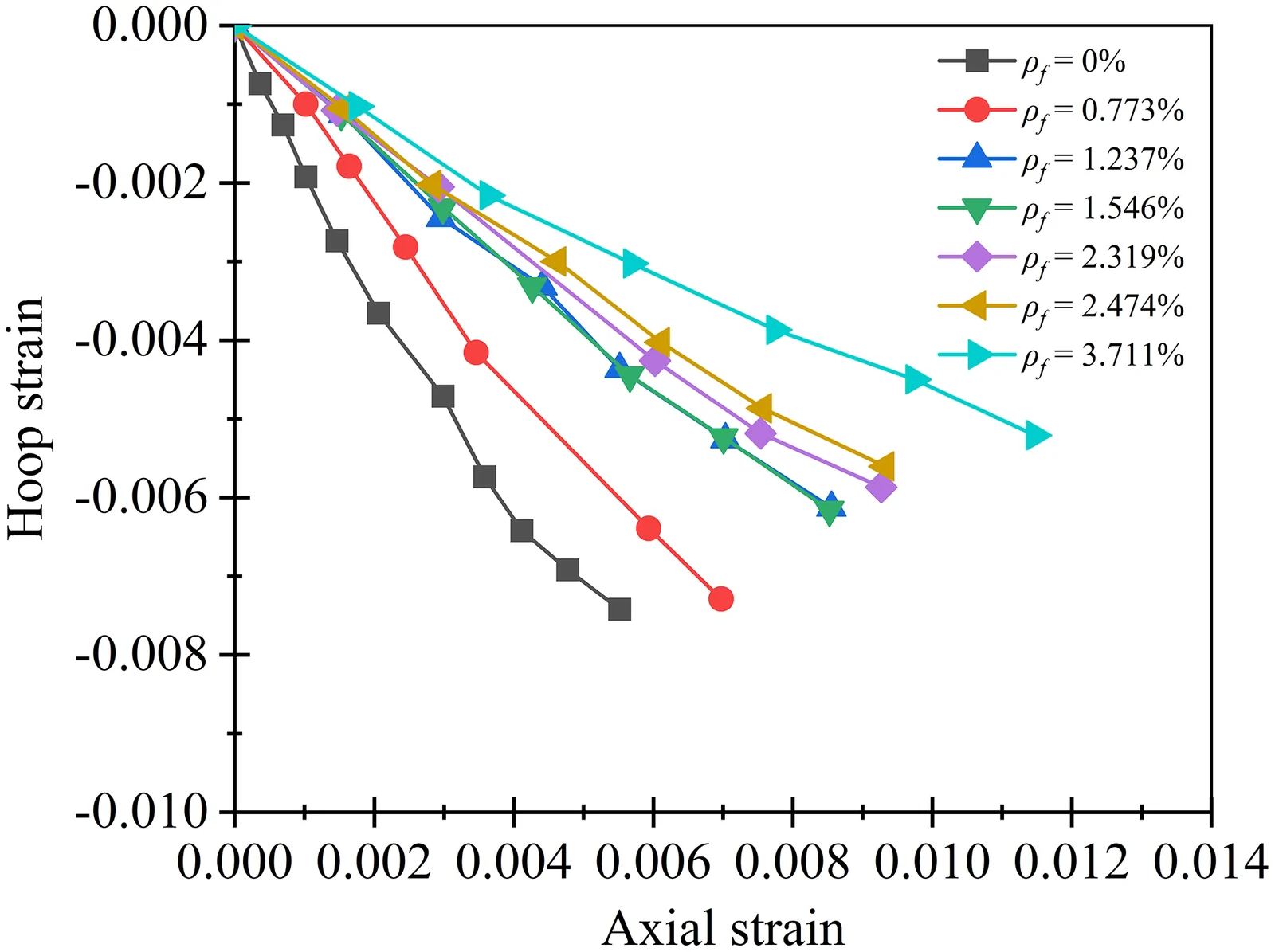
Axial strain-hoop strain curves of columns.

Fig 13 shows the correlation between axial strain and circumferential strain for externally wound CFRP composite columns, where $\rho_{f}$ denotes the CFRP volume fraction [30]. It can be seen that as $\rho_{f}$ increases, the amount of expansion decreases. When the axial strain is 0.004, columns with higher $\rho_{f}$ values exhibit reductions in circumferential strain of 23.9%, 45.7%, 47.2%, 52.5%, 54.1%, and 67.5%, respectively. Furthermore, when $\rho_{f}$ is 1.237% and 1.546%, the expansion trends of the columns are very similar, indicating comparable confinement effects for the two wrapping schemes: 1 layer at 25 mm spacing and 2 layers at 40 mm spacing.

### 3.4. Axial compressive capacity

Table 5 summarizes the test outcomes for all 20 columns, where the definitions of ultimate strength ($N_{u}$), yield strength ($N_{y}$), ultimate displacement ($\Delta_{u}$), and yield displacement ($\Delta_{y}$) are also shown in Fig 11. In order to examine how various parameters influence the capacity of columns, the reinforcement coefficient is expressed as follows [31]:

**Table 5:** Test results of columns.

Specimen	$N_{u}$ (kN)	$N_{u,aver}$ (kN)	$K_{c}$	$K_{l}$	$N_{y}$ (kN)	$N_{y,aver}$ (kN)	$\Delta_{u}$ (mm)	$\Delta_{u,aver}$ (mm)	$\Delta_{y}$ (mm)	$\Delta_{y,aver}$ (mm）	μ
CFST1	772	778	1.000	—	461	467	14.2	14.5	6.7	6.5	2.24
CFST2	784				473		14.7		6.3		
PW40-F1(1)-0	815	818	1.051	1.000	488	491	16.0	15.5	6.4	6.5	2.51
PW40-F1(2)-0	821				494		15.0		6.6		
PW40-F3(1)-0	960	966	1.242	—	577	580	17.8	17.5	6.4	6.5	2.69
PW40-F3(2)-0	971				583		17.2		6.6		
PW25-F1(1)-0	847	848	1.090	—	510	511	16.6	16.1	6.3	6.1	2.66
PW25-F1(2)-0	849				512		15.6		5.9		
PW25-F3(1)-0	1032	1034	1.329	—	617	619	19.0	19.2	6.3	6.5	3.15
PW25-F3(2)-0	1036				621		19.4		6.7		
PW25-F2(1)-10	930	932	—	—	557	559	17.1	17.2	6.3	6.0	2.83
PW25-F2(2)-10	934				561		17.3		5.7		
PW40-F2(1)-10	862	865	—	—	516	519	15.5	16.5	6.2	6.1	2.75
PW40-F2(2)-10	868				522		17.5		6.0		
PW40-F1(1)-10	831	838	—	1.024	500	502	13.9	14.9	6.1	5.9	2.34
PW40-F1(2)-10	845				504		15.9		5.7		
PW40-F1(1)-20	890	892	—	1.090	533	535	12.8	13.3	5.8	6.0	2.16
PW40-F1(2)-20	894				537		13.8		6.2		
PW40-F1(1)-30	852	840	—	1.027	511	504	12.3	12.6	5.6	5.7	2.21
PW40-F1(2)-30	828				497		12.8		5.7		

Note: $N_{u,aver}$ —Mean value of $N_{u}$; $K_{c}$ —Enhancement coefficient; $K_{l}$ —LS influence coefficient; $N_{y,aver}$ —Mean value of $N_{y}$; $\Delta_{u,aver}$ —Mean value of $\Delta_{u}$; $\Delta_{y,aver}$ —Mean value of $\Delta_{y}$; μ —Ductility coefficient


$$\begin{array}{c} K_{c}=\frac{N_{u}^{c}}{N_{u}^{\text{0}}} \end{array}$$ (1)

Where, $N_{u}^{c}$ is the ultimate strength of CFST columns confined by CFRP, $N_{u}^{\text{0}}$ is the ultimate strength of bare CFST columns. In addition, the definition of LS influence coefficient is:


$$\begin{array}{c} K_{l}=\frac{N_{u}^{L}}{N_{u}^{co}} \end{array}$$ (2)

Where $N_{u}^{L}$ is the ultimate strength of LSCFST columns confined by CFRP, and $N_{u}^{co}$ is the ultimate strength of CFRP-confined CFST columns with the same ring spacing and layer number.

**Fig 14:**
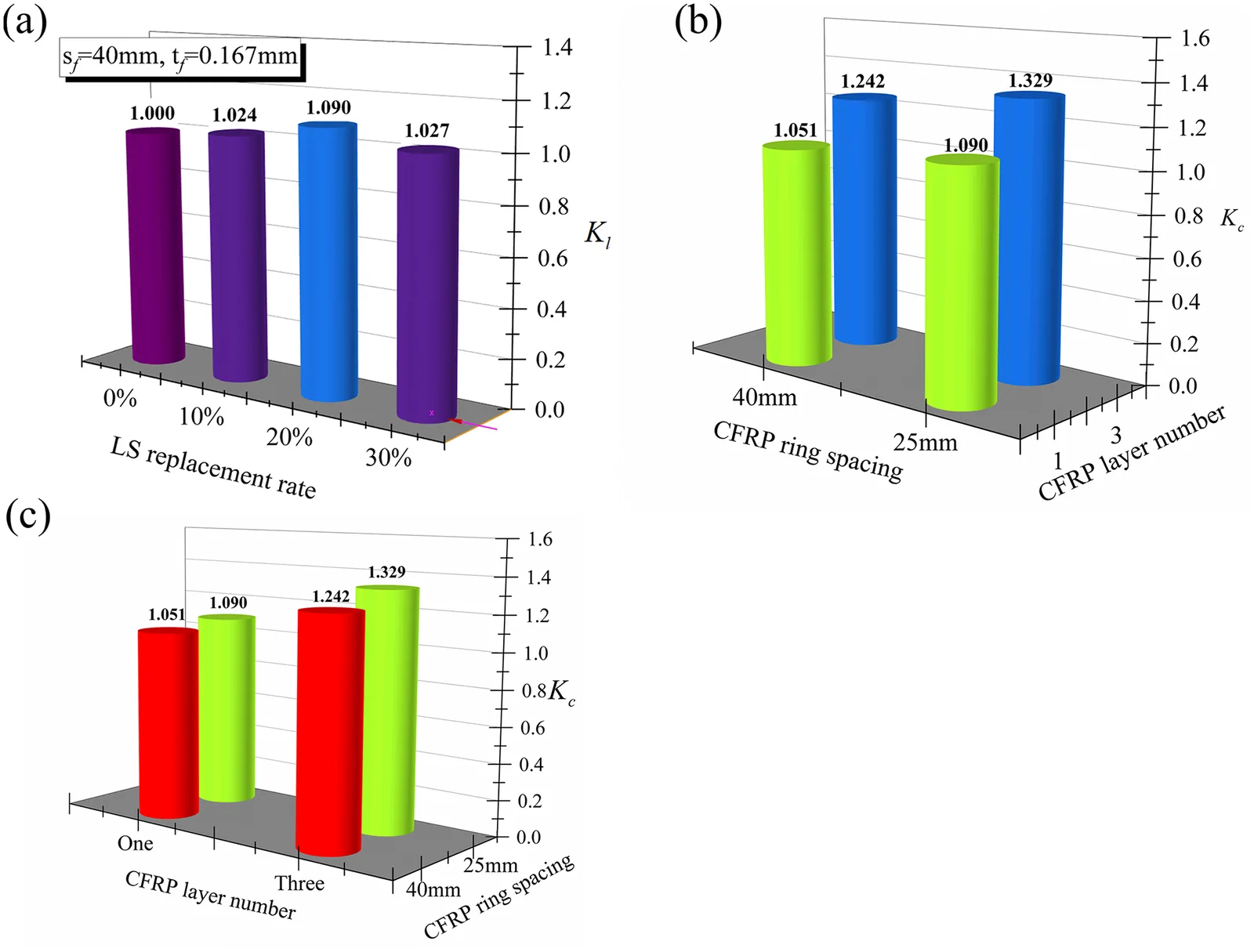
Ultimate strength of columns with different parameters. (a) LS replacement rate (b) CFRP ring spacing. (c) Number of CFRP strip layers.

Fig 14(a) illustrates the role of the LS replacement rate on the ultimate strength of columns. For PW40-F1-0, PW40-F1-10, PW40-F1-20 and PW40-F1-30 with ring spacing of 40 mm and one CFRP layer, 20% is the most suitable LS content for the load capacity of columns. When the replacement rate of LS rises from 0% to 10%, the ultimate strength increases by 2.4%. When the replacement rate of LS rises from 10% to 20%, the ultimate strength increases by 6.4%. However, the $K_{l}$ of columns decreases by 0.063 as the LS content rises to 30%. When the replacement rate is below 10%, the role of LS content on the ultimate strength of the column is limited. When the replacement rate is above 10%, adding LS significantly enhances the compressive strength of core concrete and the bearing capacity of the column.

For specimens with one CFRP layer and 40 mm ring spacing, the ultimate axial load increased when the LS replacement ratio increased from 0% to 20%, and the specimen with 20% LS replacement showed the highest capacity. This improvement may be related to the micro-filling effect and potential secondary hydration of lithium slag. However, when the LS replacement ratio increased to 30%, the axial capacity decreased, which may be attributed to the dilution effect of cementitious clinker and the increased water demand of the mixture.

Fig 14(b) reveals that the larger spacing of CFRP rings, the weaker lateral restraint it provides, resulting in reduced bearing capacity of columns. The ultimate strength of PW40-F1-0 is 818kN, and the ultimate strength of PW25-F1-0 is 848kN. When the spacing of CFRP rings reduces from 40 mm to 25 mm, the ultimate strength of columns increases by 3.7%. And the ultimate strength of PW40-F2-20 is 865kN, and the ultimate strength of PW25-F2-20 is 932kN. The spacing changes from 40 mm to 25 mm, and the ultimate strength of columns has increased by 7.5%. Moreover, the ultimate strength of PW40-F3-0 is 966kN, and the ultimate strength of PW25-F3-0 is 1034kN. The ultimate strength of columns has risen by 8.0% as CFRP ring spacing narrows.

As seen in Fig 14 (c), when the CFRP layer number added from 1 to 3, the ultimate strength of columns rose by 18.1%. And the ultimate strength of PW40-F1-10 is 838kN, and the ultimate strength of PW40-F2-10 is 865kN. As CFRP ring layers used to confine columns have increased from 1 layer to 2 layers, the ultimate strength of columns has increased by 3.5%. In addition, the ultimate strength of PW25-F1-0 is 848kN, and the ultimate strength of PW25-F3-0 is 1034kN, indicating a 21.9% gain as the CFRP layers are increased from 1 to 3. These observations clearly demonstrate that adding more CFRP layers greatly enhances the ultimate load‑bearing capacity of columns.

To evaluate the repeatability of the test results, the coefficient of variation of the duplicate ultimate axial loads was calculated. The coefficient of variation ranges from 0.17% to 2.02%, with an average value of 0.72%, indicating good repeatability of the duplicate specimens. Nevertheless, the number of repeated specimens is still limited, and a larger test database is required for more rigorous statistical evaluation.

### 3.5. Ductility coefficient

The ductility coefficient is adopted to describe the ductility of CFRP ring confined LSCFST columns, which is defined as follows:


$$\begin{array}{c} \mu =\frac{\Delta_{u}}{\Delta_{y}} \end{array}$$ (3)

**Fig 15:**
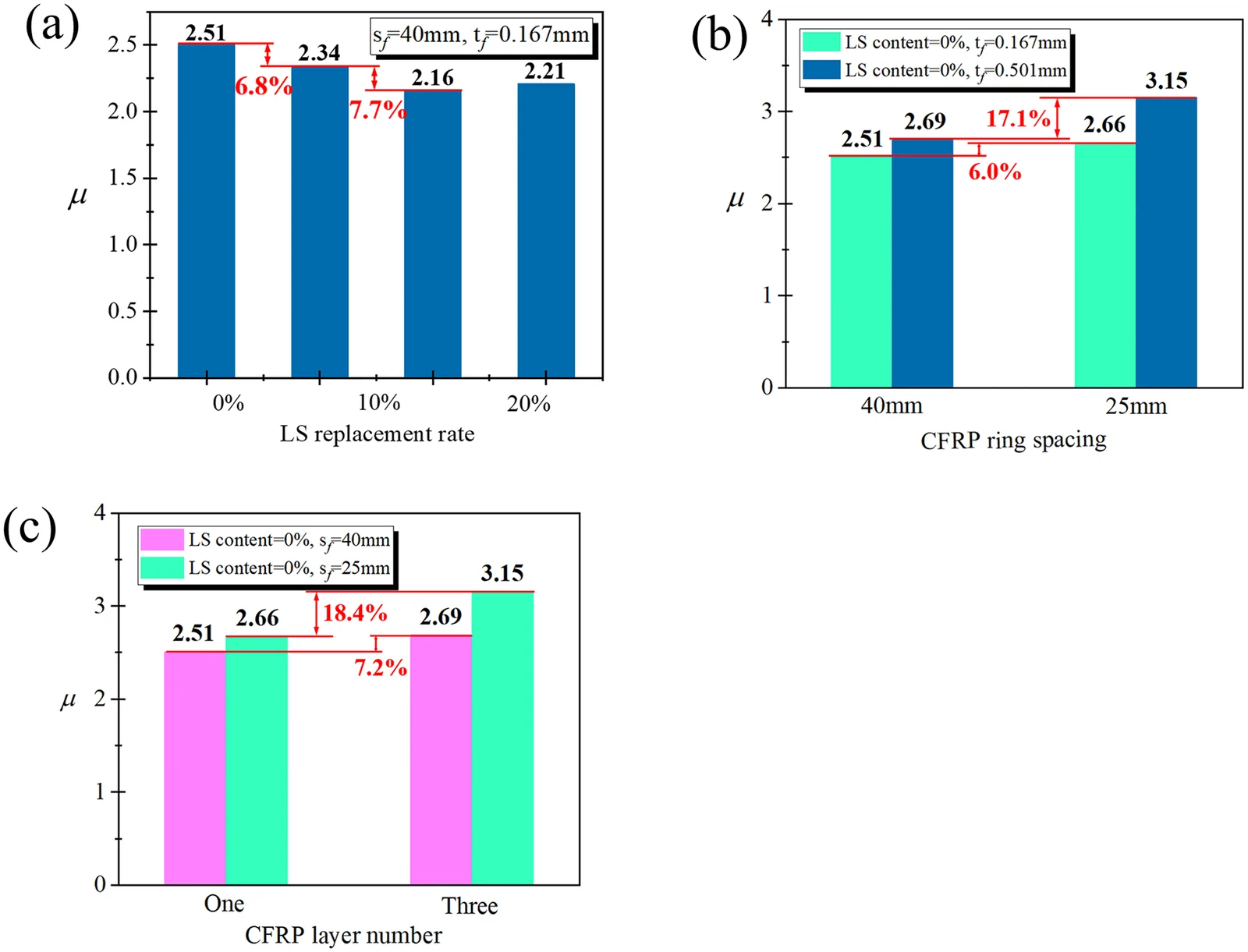
Ductility of columns with different parameters. (a) LS replacement rate. (b) CFRP ring spacing. (c) Number of CFRP strip layers.

Fig 15(a) shows the impact of LS replacement rate on ductility of CFST columns wrapped with CFRP rings. The ductility coefficients of PW40-F1-0, PW40-F1-10, PW40-F1-20, and PW40-F1-30 are 2.51, 2.34, 2.16 and 2.21, respectively. The increase in the replacement rate of LS from 0% to 10% results in a decrease in ductility of 6.7%. When LS content increased from 10% to 20%, the ductility decreased by 7.7%. Nevertheless, the μ of columns increases by 2.3% while LS content is 30%. These results show that, for columns with 40 mm spacing of one layer of CFRP rings, the addition of LS powder improves the bearing capacity but reduces the ductility of columns within 20% of LS content. The value of ductility coefficients of columns is all above 2.0, which means good seismic resistance.

Fig 15(b) shows the effect of CFRP ring spacing on column ductility. Compared with PW40-F1-0, the ductility of PW25-F1-0 columns has increased by 6.0%; Based on PW40-F2-10, PW25-F2-10 has a 3.0% increase in ductility. Compared to PW40-F3-0, PW25-F3-0 has a 17.1% increase in ductility. Under the same other parameters, the ductility deformation ability of columns is significantly enhanced by the decrease of CFRP wrapping spacing.

Fig 15(c) presents how the count of CFRP layers influences the ductility of LSCFST columns externally confined by CFRP. Compared with PW40-F1-0, the ductility of PW40-F3-0 columns increased by 7.2%. Compared to PW40-F1-10, PW40-F2-10 has a 16.5% increase in ductility. Similarly, compared to PW25-F1-0, the ductility of columns PW25-F3-0 increases by 18.5%. It indicates that adding more CFRP layers leads to an improvement in column ductility. CFRP confined columns have excellent deformation ability influenced by the thickness of CFRP.

### 3.6. Mechanism and comparison with previous CFRP-confined CFST studies

The strength development of the LS concrete core is closely related to the dosage-dependent role of LS in the cementitious matrix. When the LS replacement ratio is moderate, fine LS particles can fill capillary pores and improve particle packing, while reactive aluminosilicate phases may contribute to secondary hydration products. These effects are consistent with the observed increase in cube strength from 32.1 MPa for ordinary concrete to 35.6 MPa for 20% LS concrete. When the replacement ratio increased to 30%, the cube strength decreased to 33.2 MPa, indicating that cement dilution and higher water demand became dominant. This explains why 20% LS replacement provided the highest axial capacity in the tested PW40-F1 series.

The observed confinement mechanism is consistent with previous CFRP-confined CFST studies: external CFRP confinement delays local outward buckling of the steel tube and enhances the axial load-carrying capacity [29]. In this study, the CFRP rings increased the axial capacity of ordinary CFST specimens by 5.1% to 32.9% depending on ring spacing and layer number. Reducing the ring spacing from 40 mm to 25 mm increased the axial capacity by 3.7% to 8.0%, while increasing the CFRP layer number produced a larger enhancement of 18.1% to 21.9%. Compared with previous studies that mainly considered ordinary concrete-filled steel tubes, the present study further demonstrates that lithium slag can be incorporated into the concrete core while maintaining or improving axial strength when the replacement ratio is properly controlled [23, 32].

## 4. Simplified formulae

To establish the formula for predicting axial strength of circular LSCFST columns confined with CFRP, the European Standards EC4 [33] is used as a reference. According to the superposition theory, the axial strength consists of two parts, concrete and steel tube:


$$\begin{array}{c} N_{\text{u}}=\frac{f_{s}}{1.1}A_{s}+\frac{{f_{c}}^{\prime }}{1.5}A_{c} \end{array}$$ (4)

$$\begin{array}{c} A_{c}=\pi {\left(\frac{D}{\text{2}}\right)}^{\text{2}} \end{array}$$ (5)

$$\begin{array}{c} A_{a}=\pi {\left(\frac{D}{\text{2}}\right)}^{\text{2}}-\pi {\left(\frac{d}{\text{2}}\right)}^{\text{2}} \end{array}$$ (6)

$$\begin{array}{c} {f_{c}}^{\prime }=0.79f_{cu} \end{array}$$ (7)

Where $f_{s}$ is the yield strength of the circular steel tube; $A_{s}$ is the cross-sectional area of the circular steel tube; ${f_{c}}^{\prime }$ is the compressive strength of cylindrical concrete; $A_{c}$ is the cross-sectional area of the core concrete; D is the diameter of the steel tube; $f_{cu}$ is the compressive strength of the cubic concrete.

Lam L [34] proposed the following expression to predict the ultimate strength of CFST members externally confined by CFRP:


$$\begin{array}{c} \frac{{f_{cc}}^{\prime }}{{f_{co}}^{\prime }}=1+k_{\text{1}}\frac{f_{l}}{{f_{co}}^{\prime }} \end{array}$$ (8)

Where ${f_{cc}}^{\prime }$ is the axial compressive strength of the confined concrete; ${f_{co}}^{\prime }$ is the axial compressive strength of unconfined concrete; $f_{l}$ is the lateral constraint compressive stress; $k_{\text{1}}$ is the effective constraint stress coefficient, and in this paper, the effective constraint stress coefficient $k_{\text{1}}=2$.

In addition, a coefficient λ is defined to account for the influence of LS content on the compressive strength of concrete. The LS coefficient can be obtained as follows:


$$\begin{array}{c} \lambda =-0.00005L{i_{per}}^{\text{2}}+0.00638Li_{per}+1 \end{array}$$ (9)

Where λ is the coefficient of LS; $Li_{per}$ is the percentage of LS content.

For the partial wrapping scheme of CFRP in this paper, a constraint influence factor of CFRP strips is proposed by Saadatmanesh [35] to obtain the lateral confining stress of CFRP strips:


$$\begin{array}{c} {f_{l,CFRP}}^{\prime }=k_{g}f_{l,CFRP} \end{array}$$ (10)

$$\begin{array}{c} f_{l,CFRP}=\frac{2f_{CFRP}t_{CFRP}}{D} \end{array}$$ (11)

$$\begin{array}{c} t_{CFRP}=0.17n \end{array}$$ (12)

$$\begin{array}{c} k_{g}=\frac{{\left(1-\frac{s_{CFRP}}{2D}\right)}^{\text{2}}}{1-\rho_{sc}} \end{array}$$ (13)

Where, ${f_{l,CFRP}}^{\prime }$ is the lateral restraint stress for partially wrapping; $k_{g}$ is the spacing coefficient of CFRP rings; $f_{l,CFRP}$ is the lateral restraint stress for fully wrapping; $t_{CFRP}$ is the thickness of wrapping CFRP rings; $s_{CFRP}$ is the net spacing between CFRP rings; $\rho_{sc}$ is the longitudinal reinforcement ratio.

Therefore, the formula for the axial compressive strength of LSCFST columns wrapped by CFRP is:


$$\begin{array}{c} {f_{cc}}^{\prime }={f_{co}}^{\prime }+k_{\text{1}}\frac{{\left(1-\frac{s_{CFRP}}{2D}\right)}^{\text{2}}}{1-\rho_{sc}}\times \frac{2f_{CFRP}\times 0.17n}{D} \end{array}$$ (14)

$$\begin{array}{c} N_{u}=\frac{f_{s}}{1.1}A_{s}+0.53\lambda {f_{cc}}^{\prime }A_{c} \end{array}$$ (15)

**Fig 16:**
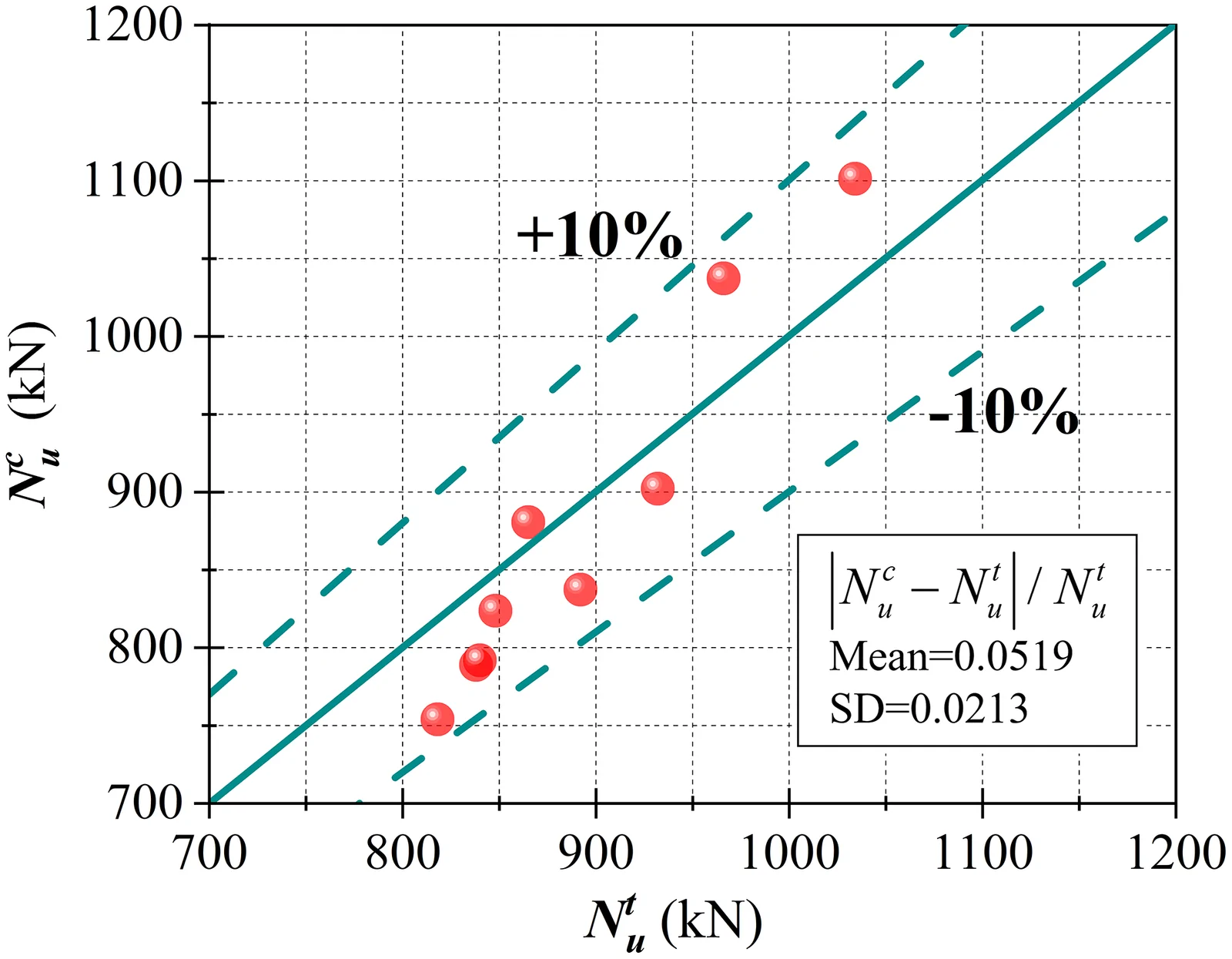
Comparison of compressive strength given by the experiment and the calculation.

The calculated value of test columns was obtained by substituting test data into this formula and contrasted with the experimental value, as listed in Table 6 and Fig 16. The range of error between the experimental and calculated values is 0.0173 ~ 0.0781, with an average value of 0.0519.

**Table 6:** Summary of the calculated and experimental results.

Specimen	$N_{u}$ (kN)	$N_{u}^{t}$ (kN)	$N_{u}^{c}$ (kN)	$\left|N_{u}^{c}-N_{u}^{t}\right|/N_{u}^{t}$ (%)
PW40-F1(1)-0	815	818	754.09	7.81
PW40-F1(2)-0	821			
PW40-F3(1)-0	960	966	1037.35	7.38
PW40-F3(2)-0	971			
PW25-F1(1)-0	847	848	823.83	2.85
PW25-F1(2)-0	849			
PW25-F3(1)-0	1032	1034	1101.36	6.42
PW25-F3(2)-0	1036			
PW25-F2(1)-10	930	932	902.28	3.19
PW25-F2(2)-10	934			
PW40-F2(1)-10	862	865	880.77	1.73
PW40-F2(2)-10	868			
PW40-F1(1)-10	831	838	788.95	5.97
PW40-F1(2)-10	845			
PW40-F1(1)-20	890	892	837.46	6.17
PW40-F1(2)-20	894			
PW40-F1(1)-30	852	840	791.87	5.73
PW40-F1(2)-30	828			

**Fig 17:**
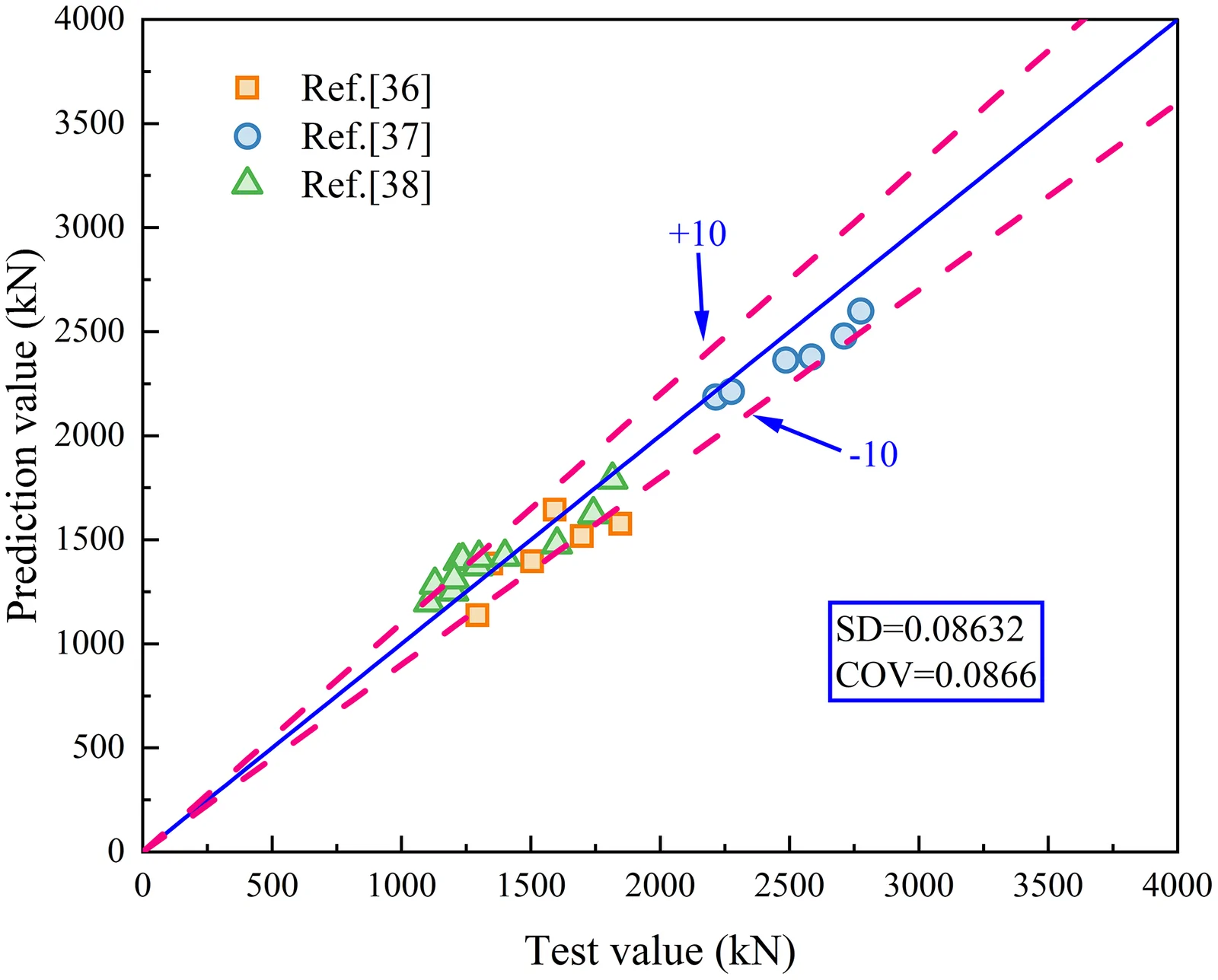
Validation of proposed model.

Due to the limited nature of the test data in this paper, external data was used to validate the proposed model’s applicability and accuracy [36–38]. Table 7 lists the various parameters for external data. Fig 17 shows a comparison of the test data and the predicted values. The results show that the predicted values are generally within a margin of error of ±10%, with a standard deviation of 0.08632 and a coefficient of variation (COV) of 0.0866. This indicates that the predictive model has good applicability and accuracy.

**Table 7:** Validation of predictive model.

Specimens	tcf(mm)	fcf(MPa)	ts(mm)	As(mm²)	fs(MPa)	fck(MPa)	Nt(kN)	Ref.
1–2.5	0.17	1260	2.5	1013.2	350	40.15	1294	[36]
1–3.5	0.17	1260	3.5	1440.4	310	40.15	1348	
1–4.5	0.17	1260	4.5	1880.2	310	40.15	1698	
2–2.5	0.34	1260	2.5	1013.2	350	40.15	1506	
2–3.5	0.34	1260	3.5	1440.4	310	40.15	1593	
2–4.5	0.34	1260	4.5	1880.2	310	40.15	1846	
SC41	0.167	1500	4	2400	295	53.6	2215	[37]
SC42	0.334	1500	4	2400	295	53.6	2275	
SC51	0.167	1500	5	3000	295	53.6	2485	
SC52	0.334	1500	5	3000	295	53.6	2585	
SC61	0.167	1500	6	3600	295	53.6	2710	
SC62	0.334	1500	6	3600	295	53.6	2775	
A-1	0.111	4900	3.5	1960	300	22.3	1107	[38]
A-2	0.222	4900	3.5	1960	300	22.3	1129	
A-3	0.333	4900	3.5	1960	300	22.3	1222	
B-1	0.111	4900	3.5	1960	300	26.4	1200	
B-2	0.222	4900	3.5	1960	300	26.4	1237	
B-3	0.333	4900	3.5	1960	300	26.4	1294	
C-1	0.111	4900	3.5	1960	300	32.8	1204	
C-2	0.222	4900	3.5	1960	300	32.8	1300	
C-3	0.333	4900	3.5	1960	300	32.8	1400	
D-1	0.111	4900	3.5	1960	300	40	1601	
D-2	0.222	4900	3.5	1960	300	40	1742	
D-3	0.333	4900	3.5	1960	300	40	1815	

## 5. Conclusion

An experiment was conducted on the axial behavior of CFRP ring-confined LSCFST columns. Test results support these conclusions:

(1) The LSCFST column wrapped FRP strips exhibits a progressive failure mode under axial compressive conditions. It fails due to outward buckling on circular steel tubes. The lateral confinement of FRP effectively delays the steel tube deformation, and improves the strength of columns.
(2) Under the same axial deformation condition, CFRP confined columns with $\rho_{f}$ =3.711% have the minimal hoop strain. And it is a similar confinement effect on columns with $\rho_{f}$ =1.237% and columns with $\rho_{f}$ =1.546% for their same dilation trend.
(3) As the rising of LS content, the $K_{l}$ of LSCFST columns wrapped with CFRP increases, but their μ deteriorates. As the spacing between CFRP rings decreases, the $K_{c}$ and μ of columns increase. As the CFRP layer number adds, $K_{c}$ and μ of columns are also enhanced.
(4) Based on researches on CFRP confinement effect and LS concrete, a prediction model for $N_{u}$ of LSCFST circular columns confined by CFRP rings is proposed. According to calculation results, the values of this model are consistent with experimental values.

Although duplicate specimens were tested for each configuration and good repeatability was obtained, the sample size remains limited. Future studies should include more repeated specimens to improve the statistical reliability of the conclusions and the proposed prediction model.

Direct SEM, XRD and pore-structure characterization should be carried out in future studies to further verify the microstructural mechanism of lithium-slag concrete in CFRP-confined LSCFST columns. Future research should investigate slender columns, eccentric compression, cyclic loading, seismic performance and long-term durability to broaden the practical applicability of CFRP ring-confined LSCFST columns.
